# Spending at Mobile Fruit and Vegetable Carts and Using SNAP Benefits to Pay, Bronx, New York, 2013 and 2014

**DOI:** 10.5888/pcd12.140542

**Published:** 2015-06-04

**Authors:** Andrew Breck, Kamila M. Kiszko, Courtney Abrams, Brian Elbel

**Affiliations:** Author Affiliations: Andrew Breck, New York University Robert F. Wagner Graduate School of Public Service and New York University School of Medicine, New York, New York; Kamila M. Kiszko, Courtney Abrams, New York University School of Medicine, New York, New York. Dr Elbel is also affiliated with New York University Robert F. Wagner Graduate School of Public Service.

## Abstract

This study examines purchases at fruit and vegetable carts and evaluates the potential benefits of expanding the availability of electronic benefit transfer machines at Green Carts. Customers at 4 Green Carts in the Bronx, New York, were surveyed in 3 waves from June 2013 through July 2014. Customers who used Supplemental Nutrition Assistance Program benefits spent on average $3.86 more than customers who paid with cash. This finding suggests that there may be benefits to increasing the availability of electronic benefit transfer machines at Green Carts.

## Objective

In 2008, New York City implemented a policy that established 1,000 permits for mobile fruit and vegetable vendors to locate in neighborhoods with the scarcest levels of healthful food (1). The goal of this initiative, Green Carts, was to introduce a low-cost mechanism to increase the consumption of fresh produce (2,3).

However, the lack of fresh produce may be only part of the obstacle to a healthy diet. Green Cart permits restrict vending locations to low-income neighborhoods, where many residents purchase food with Supplemental Nutrition Assistance Program (SNAP) benefits. Financial support from New York State Department of Health beginning in 2010 covered the $900 cost of an electronic benefit transfer (EBT) machine necessary to accept SNAP benefits and the first 3 months of fees ($35/month plus 3.5 cents/transaction) for eligible vendors. Even after the implementation of this program, less than a third of vendors were equipped with EBT machines (4–6).

To evaluate the possible benefits of expanding the introduction of EBT machines at produce carts, we examined whether consumers spend more on fruits and vegetables per transaction at Green Carts when they pay with SNAP benefits than when they pay with cash.

## Methods

Customers at 4 Green Carts in the Bronx, New York, were surveyed in 3 waves: June–July 2013, September–October 2013, and June–July 2014. The New York City Department of Health and Mental Hygiene identified 4 vendors as responsive and amenable to participating in the study. Two carts were equipped with EBT machines, and 2 more carts were expected to receive them shortly after our first data collection period. Green Cart customers who appeared to be adults (aged ≥18 y) were invited to voluntarily participate in a brief survey about their shopping behaviors and a “bag check” to determine what items the participant purchased. Surveys were conducted in either Spanish or English on 29 weekdays between 1:30 PM and 5:30 PM. During each round of data collection, we collected approximately 100 surveys at participating carts. Our overall response rate was 70%. Respondents received a transit pass valued at $2.50 upon completion of the survey and bag check. This study was approved by New York University School of Medicine’s institutional review board.

Mean dollars spent per transaction and frequencies of demographic variables were calculated for each of 2 segments of the survey sample: 1) for the entire sample of respondents and 2) for only the consumers surveyed at Green Carts equipped with an EBT machine. Controlling for customer characteristics that we hypothesized are associated with fresh produce purchases, including race/ethnicity, age, sex, education, and annual household income, we used linear regression models to calculate separately the association between payment method and the amount spent on Green Cart purchases for each analytic sample. Analyses were conducted using Stata version 12 (StataCorp LP).

## Results

The full sample included 782 transactions at 4 Green Carts (Table 1). Most respondents were women (74.2%), were Hispanic (53.7%), had no more than a high school degree (63.7%), and lived in a household with an annual income of less than $25,000 (53.6%). Respondents were approximately equally likely to report that they usually purchased fruits and vegetables at a grocery store (41.7%) as they were to report buying these items from a produce cart (44.9%). On average, consumers spent $4.19 per transaction at the Green Cart. Most paid for their purchase using only cash (87.3%); 41.9% reported receiving SNAP benefits.

**Table 1:** Descriptive Statistics for Full Sample and Subsamples of Survey Respondents at 4 Green Carts in the Bronx, New York, 2013 and 2014^a^

Characteristic	Entire Sample (n = 782)	Consumers at Green Carts With EBT (n = 516)	*P* Value for Association Between Subsample and Rest of Sample^b^	Consumers Who Made Purchases Using SNAP Benefits (n = 99)	*P* Value for Association Between Subsample and Rest of Sample^b^
**Spent at Green Cart, mean (SD), $**	4.19 (3.85)	4.54 (4.12)	<.001	8.20 (5.56)	<.001
**Payment method**					
Cash only	683 (87.3)	417 (80.8)	<.001	0	<.001
SNAP or SNAP and cash	99 (12.7)	99 (19.2)		99 (100.0)	
**Sex**					
Male	202 (25.8)	110 (21.3)	<.001	10 (10.1)	<.001
Female	580 (74.2)	406 (78.7)		89 (89.9)	
**Race/ethnicity**					
White	44 (5.6)	22 (4.3)	<.001	7 (7.1)	<.001
Black	197 (25.2)	113 (21.9)		9 (9.1)	
Hispanic	420 (53.7)	304 (58.9)		74 (74.8)	
Other or refused	121 (15.5)	77 (14.9)		9 (9.1)	
**Age, y**					
18–24	26 (3.3)	19 (3.7)	.42	5 (5.1)	.006
25–39	257 (32.9)	178 (34.5)		46 (46.5)	
40–64	402 (51.4)	258 (50.0)		41 (41.4)	
≥65	97 (12.4)	61 (11.8)		7 (7.1)	
**Education**					
High school degree or less	498 (63.7)	359 (69.6)	<.001	77 (77.8)	.02
Some college	138 (17.7)	80 (15.5)		13 (13.1)	
BA or more	105 (13.4)	53 (10.3)		7 (7.1)	
Missing or refused	41 (5.2)	24 (4.7)		2 (2.0)	
**Annual household income, $**					
<25,000	419 (53.6)	300 (58.1)	<.001	76 (76.8)	<.001
25,000–49,999	175 (22.4)	100 (19.4)		8 (8.1)	
≥50,000	81 (10.4)	43 (8.3)		2 (2.0)	
Missing or refused	107 (13.7)	73 (14.2)		13 (13.1)	
**Employment status**					
Not employed	285 (36.5)	203 (39.3)	.02	63 (63.6)	<.001
Retired	102 (13.0)	58 (11.2)		7 (7.1)	
Working, part- or full-time	395 (50.5)	255 (49.4)		29 (29.3)	
**SNAP recipient**					
No	454 (58.1)	276 (53.5)	<.001	3 (3.0)	<.001
Yes	328 (41.9)	240 (46.5)		96 (97.0)	
**Usual source for fruits and vegetables**					
Grocery store	326 (41.7)	185 (35.9)	<.001	27 (27.3)	<.001
Produce cart	351 (44.9)	263 (51.0)		64 (64.7)	
Farmers market, produce store, or bodega	59 (7.5)	34 (6.6)		3 (3.0)	
Don’t know or other	46 (5.9)	34 (6.6)		5 (5.1)	
**Vendor**					
A	182 (23.3)	95 (18.4)	<.001	9 (9.1)	<.001
B	180 (23.0)	180 (34.9)		52 (52.5)	
C	241 (30.8)	241 (46.7)		38 (38.4)	
D	179 (22.9)	NA		NA	
**How often respondent shops at Green Cart**					
<2 or 3 times/month	176 (22.5)	91 (17.6)	<.001	14 (14.1)	.01
1-6 times/week	460 (58.8)	337 (65.3)		72 (72.7)	
At least once/day	146 (18.7)	88 (17.1)		13 (13.1)	

Abbreviations: BA, Bachelor of Arts; EBT, electronic benefits transfer; NA, not applicable; SD, standard deviation; SNAP, Supplemental Nutrition Assistance Program.

a Values are number (percentage) unless otherwise indicated.

b Two-sided *t* test was used for dollars spent at Green Cart; χ^2^ used for all other categories.

At EBT-equipped carts, 19.2% of respondents reported using SNAP benefits (or SNAP and cash) to pay for their purchase. Customers who reported using SNAP benefits at a Green Cart spent more money on fruits and vegetables: $8.20, on average per transaction, compared with cash-only customers who spent $3.68, on average per transaction at EBT-equipped Carts. Customers using SNAP benefits at Green Carts were more likely to report that they usually purchase fruits and vegetables from produce carts (64.7%) than from any other source, including grocery stores (27.3%). Most survey respondents who reported using SNAP at Green Carts were Hispanic (74.8%), women (89.9%), not employed (63.6%), or living in households with less than $25,000 in annual income (76.8%).

Linear regression showed a significant, large, and positive association between the use of SNAP benefits compared with cash at Green Carts and the total amount spent per transaction (Table 2). The results were robust across regressions on the entire sample ($3.86, *P* < .001) and on the sample restricted to consumers at Green Carts equipped with an EBT machine ($3.81, *P* < .001).

**Table 2:** Linear Regression Results of Dollars Spent at 4 Mobile Fruit and Vegetable Carts in the Bronx, New York, 2013 and 2014

Characteristic	All Survey Respondents (n = 782)		Only Respondents Shopping at Green Carts With an EBT Machine (n = 516)	
	β	*P* Value	β	*P* Value
**Payment method**				
Cash only	—	—	—	—
SNAP or SNAP and cash	3.861	<.001	3.812	<.001
**Sex**				
Male	—	—	—	—
Female	0.53	.07	0.823	.04
**Race/ethnicity**				
White	—	—	—	—
Black	0.588	.31	0.432	.60
Hispanic	1.191	.03	0.922	.24
Other or refused	0.848	.16	1.03	.23
**Age, y**				
18–24	—	—	—	—
25–39	0.767	.28	1.802	.04
40–64	0.191	.79	0.839	.33
≥65	−0.096	.91	0.541	.61
**Education**				
High school degree or less	—	—	—	—
Some college	−0.443	.19	−0.025	.96
BA or more	0.233	.56	−0.065	.91
Missing or refused	0.393	.50	1.174	.14
**Annual household income, $**				
<25,000	—	—	—	—
25,000–49,999	−0.453	.18	−0.460	.30
≥50,000	0.418	.38	−0.587	.37
Missing or refused	0.011	.98	−0.528	.26
**Employment status**				
Not employed	—	—	—	—
Retired	0.620	.23	−0.022	.97
Working, part- or full-time	0.317	.28	−0.245	.51
**SNAP recipient**				
No	—	—	—	—
Yes	−0.143	.64	−0.569	.15
**Usual source for fruits and vegetables**				
Grocery store	—	—	—	—
Produce cart	0.366	.19	0.295	.40
Farmers market, produce store, or bodega	0.342	.48	−0.330	.61
Don’t know or other	−0.445	.42	−0.603	.38
**Vendor**				
A	—	—	—	—
B	1.987	<.001	2.087	<.001
C	−0.724	.09	−0.44	.33
D	0.560	.22	NA	NA
**How often respondent shops at this green cart**				
<2 or 3 times/month	—	—	—	—
1–6 times/week	0.236	.46	0.597	.17
At least once/day	−0.213	.60	0.020	.97
**Vendor accepts EBT**				
No	—	—	NA	NA
Yes	0.066	.90	NA	NA
**Constant**	1.285	.19	0.824	.52

Abbreviations: BA, Bachelor of Arts, EBT, electronic benefits transfer; NA, not applicable; SNAP, Supplemental Nutrition Assistance Program (SNAP).

## Discussion

Customers who used SNAP benefits at EBT-equipped Green Carts in the Bronx, New York, spent on average $3.81 more than customers who paid with cash. This study has several limitations. Sales and profit data were not available. The 4 Green Carts were located in only 1 borough of New York City and surveys were collected only during select times of day, so we do not know the extent to which the people we surveyed represent all Green Cart customers. Furthermore, we lacked information on fresh produce purchases made from other retailers, and thus we do not know whether SNAP users were purchasing more fruits and vegetables in total.

There are likely multiple barriers for households to access fresh food. Because the residents of areas with low availability of fresh produce are predominantly low income, the introduction of Green Carts to the neighborhood may be a first step toward increasing fruit and vegetable consumption. It is then possible that the availability to pay with SNAP benefits might result in increased expenditures at Green Carts, helping to overcome a barrier to a healthy diet. We do not claim that our findings are necessarily causal, nor do we assess the effect of this increased spending on nutritional quality. The results from our analysis suggest there may be benefits to introducing EBT machines to produce carts, suggesting the policy could be sustainably scalable to other urban areas, although more research is necessary to identify causal effects that justify making recommendations for policy makers.
